# A hyperbranched dopamine-containing PEG-based polymer for the inhibition of α-synuclein fibrillation

**DOI:** 10.1016/j.bbrc.2015.12.060

**Published:** 2016-01-22

**Authors:** Leonid Breydo, Ben Newland, Hong Zhang, Anne Rosser, Carsten Werner, Vladimir N. Uversky, Wenxin Wang

**Affiliations:** a Department of Molecular Medicine and Byrd Alzheimer's Research Institute, Morsani College of Medicine, University of South Florida, 33612 Tampa, FL, USA; b Leibniz-Institute für Polymerforschung, Dresden, Germany; c Brain Repair Group, School of Biosciences, Cardiff University, Cardiff, UK; d The Charles Institute of Dermatology, School of Medicine and Medical Science, University College Dublin, Dublin, Ireland; e Institute for Biological Instrumentation, Russian Academy of Sciences, 142292 Pushchino, Moscow Region, Russia; f Department of Biological Sciences, Faculty of Science, King Abdulaziz University, PO Box 80203, 21589 Jeddah, Saudi Arabia; g School of Materials Science and Engineering, Tianjin University, Tianjin, China

**Keywords:** Hyperbranched polymers, Dopamine, Macromolecular crowding, Parkinson's disease, Protein aggregation, RAFT polymerization, α-synuclein, DOPA, dopamine, DP, dopamine-containing polymer, PEG, polyethylene glycol, ThT, thioflavin T, RAFT, reversible addition-fragmentation chain transfer

## Abstract

Aggregation of α-synuclein is believed to play an important role in Parkinson's disease and in other neurodegenerative maladies. Small molecule inhibitors of this process are among the most promising drug candidates for neurodegenerative diseases. Dendrimers have also been studied for anti-fibrillation applications but they can be difficult and expensive to synthetize. Here we show that RAFT polymerization can be used to produce a hyperbranched polyethylene glycol structure via a one-pot reaction. This polymer included a dopamine moiety, a known inhibitor of α-synuclein fibril formation. Dopamine within the polymer structure was capable of aggregation inhibition, although not to the same degree as free dopamine. This result opens up new avenues for the use of controlled radical polymerizations as a means of preparing hyperbranched polymers for anti-fibrillation activity, but shows that the incorporation of functional groups from known small molecules within polymers may alter their biological activity.

## Highlights

• Dopamine-containing hyperbranched polymer was synthesized by RAFT co-polymerization.
• This polymer inhibited α-synuclein aggregation less effectively than dopamine.
• It was also much less effective than dopamine in disaggregating α-synuclein fibrils.
• The difference is likely due to loss of covalent dopamine–protein interactions.

## Graphical abstract

**Figure:**
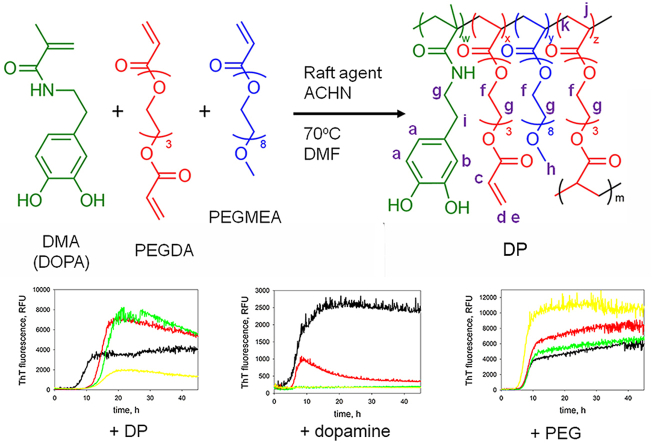

## 1 Introduction

The molecular basis of Parkinson's disease (PD) appears to be tightly coupled to the aggregation of α-synuclein. Autosomal dominant early-onset PD is induced as a result of six different missense mutations in the α-synuclein gene [1], [2], [3], [4] or as a result of the overexpression of the wild type α-synuclein protein due to gene triplication [5], [6], [7]. In addition, aggregates of α-synuclein were found to be the major components of Lewy bodies and Lewy neurites, the hallmarks of PD [8], [9], [10], [11], [12]. These *in vivo* results have been supported by numerous studies that established that α-synuclein aggregates into amyloid fibrils and oligomers under a variety of conditions including physiological [13], [14], [15].

In recent years, polymers and nanoparticles have been explored, not only for the more established field of protein aggregate detection, diagnosis and destruction [16], [17], but also for the study of protein fibril formation [18] and the prevention of fibrillation [19], [20]. Polymers have a variety of effects on protein fibrillation, as some of them have been shown to accelerate fibrillation [18], [21], yet others retard the fibrillation process [22]. Dendrimers are a sub-class of polymers, which have a symmetrical and well-ordered tree-like structure. The high degree of control over the exact structure has led to extensive investigation of their use as drug delivery agents [23], gene delivery vectors [24], and, more recently, as molecules to inhibit the fibrillation of α-synuclein [25]. However, dendrimers are synthesized via a complex step-wise growth process, which requires purification after each step and is therefore costly. Alternatively, living polymerizations such as deactivation-enhanced atom transfer radical polymerization and reversible addition–fragmentation chain transfer (RAFT) have recently been shown to be capable of allowing highly branched (or hyperbranched) soluble polymeric structures [26], or cyclized structures [27], [28] via simple one-pot reactions. Furthermore, they easily allow the inclusion of an extensive range of functional monomers in the co-polymerization reactions.

In this study, we show the proof-of-principle, that RAFT polymerization can be used to produce a hyperbranched poly (ethylene glycol) (PEG) structure which contains a DOPA moiety hypothesized to provide anti-fibrillation properties. We aimed to investigate whether the inclusion of a DOPA molecule into a hyperbranched structure would reduce, enhance or have no effect on anti-fibrillation or aggregation behavior. For the synthesis of this functionalized polymer, a DOPA analogue with a methacrylamide group (to allow incorporation in living radical polymerizations) was firstly synthesized and incorporated into a hyperbranched PEG via co-polymerization. This polymer, DOPA-PEG polymer (henceforth termed DP) was analyzed at different concentrations to assess its effect on α-synuclein aggregation/fibrillation *in vitro*. We found that, similar to dopamine itself, DOPA-modified polymer interfered with the α-synuclein fibril formation promoting oligomer formation instead. However, effects of dopamine were significantly moderated by its incorporation into a polymer, and it was no longer capable of effectively disaggregating fibrils into oligomers. The ease at which the polymer structure and composition can be varied allows the potential to mechanistically study specific polymer/protein interactions in an attempt to find future therapeutic strategies for diseases associated with protein misfolding.

## 2 Materials and methods

### 2.1 Materials

Dopamine hydrochloride, sodium bicarbonate, methacrylate anhydride, sodium borate, sodium hydroxide, magnesium sulfate, poly(ethylene glycol) methyletheracrylate (PEGMEA, Mn = 575 gmol^-1^), poly(ethylene glycol) diacrylate (PEGDA, Mn = 258 gmol^-1^), 2,2-dimethoxy-2-phenylacetophenone and 1,10-Azobis-cyclohexane-carbonitrile (ACHN) were purchased from Sigma. Acetone 99.8+%, tetrahydrofuran, hexane 95%, dimethylformamide (DMF), ethyl acetate, hydrochloric acid, and methanol were purchased from Fisher Scientific. 4-Cyano-4-[(ethylsulfanylthiocarbonyl)sulfanyl] pentanoic acid was obtained as a kind gift from Dr. Hongyun Tai at Bangor University, UK. Other chemicals and supplies were from Sigma, Fisher or VWR.

### 2.2 Polymer synthesis and characterization

The dopamine methacrylamide (DMA) monomer was prepared via a previously reported protocol [29], and characterized as reported previously [30]. The DP polymer was synthesized by RAFT polymerization using ACHN as an initiator and 4-Cyano-4-[(ethylsulfanylthiocarbonyl) sulfanyl] pentanoic acid as the RAFT agent as described previously [30]. The solvent (DMF) and reagents were added to a 100 mL round bottomed flask in the following mole ratio: ACHN/Raft agent/DMA/PEGMEA/PEGDA = 1: 2: 40: 40: 20 respectively. Oxygen was removed by bubbling nitrogen through the rubber stopped sealed flask for 20 min. After 17 h reaction time in at 70 °C (stirring at 700 rpm), the reaction was terminated by exposing it to the air. The polymer was purified by dilution in methanol followed by precipitation in diethyl ether, then dilution in methylene chloride and further precipitation in diethyl ether. The viscous brown polymer was finally dried in a vacuum oven. Gel permeation chromatography (Agilent, PL-GPC50 with RI detector) was used to analyze the polymer molecular weight as calibrated by poly (methyl methacrylate) standards. ^1^H NMR was performed using a 400 MHz Bruker NMR with Delta NMR processing software with the chemical shifts referenced to chloroform (CDCl_3_).

### 2.3 Protein aggregation assays

Protein aggregation in the automated format was carried out in a reaction volume of 0.1 mL in black, flat-bottomed 96-well plates in the presence of 5 μM thioflavin T (ThT). Two teflon balls (2.38 mm diameter, Precision Ball, Reno, PA) were placed into each well of a 96-well plate. The reaction mixture containing protein and ThT (320 μl) was split into three wells (100 μl into each well), the plates were covered by Mylar septum sheets (Thermo), and incubated at 40 °C with continuous orbital shaking at 280 rpm in an Infinite M200 Pro microplate reader (Tecan, Austria). Reaction conditions: 0.25 mg/mL (17.4 μM) α-synuclein in 20 mM Hepes buffer (pH 7.5), 100 mM NaCl, 5 μM ThT and 50 μg/mL heparin. The kinetics was monitored by top reading of fluorescence intensity every 5 min using 444 nm excitation and 485 nm emission filters. Data from replicate wells were averaged before plotting fluorescence vs. time. The data were fit to a sigmoidal equation (Eqn. (1)) using SigmaPlot (Systat, San Jose, CA).

(1) $$\text{F}=\text{A}+\text{B}/\left(1+\exp\left(\text{k}\times \left(\text{t}-{\text{t}}_{\text{m}}\right)\right)\right)$$

Here A is the initial level of ThT fluorescence, B is the difference between the final level of ThT fluorescence and its initial level, k is the rate constant of amyloid accumulation (h^−1^), and t_m_ is the midpoint of transition. The lag time (t_l_) of amyloid formation was calculated as t_l_ = t_m_−2/k. Initiation rate was defined as the inverse of lag time.

Fibril disaggregation assays were conducted in a similar manner except preformed α-synuclein fibrils (0.05 mg/mL) were incubated (20 mM Hepes buffer, pH 7.5, 100 mM NaCl, 5 μM ThT and 50 μg/mL heparin, 40 °C) in the presence of variable concentrations of either dopamine or a dopamine-containing polymer (DP) in the plate reader with shaking, and ThT fluorescence was monitored. Fibril disaggregation was analyzed using the three parameter exponential decay equation using SigmaPlot (Systat, San Jose, CA).

### 2.4 Electron microscopy

5 μl aliquot of the protein solution was adsorbed onto prewashed 200 mesh formvar/carbon-coated nickel grids for 5 min. The grid was washed with water (10 μl), stained with 2% uranyl acetate (10 μl) for 2 min and washed with water again. The samples were analyzed with a JEM 1400 transmission electron microscope (JEOL) operated at 80 kV.

## 3 Results and discussion

### 3.1 Polymer synthesis

**Fig. 1:**
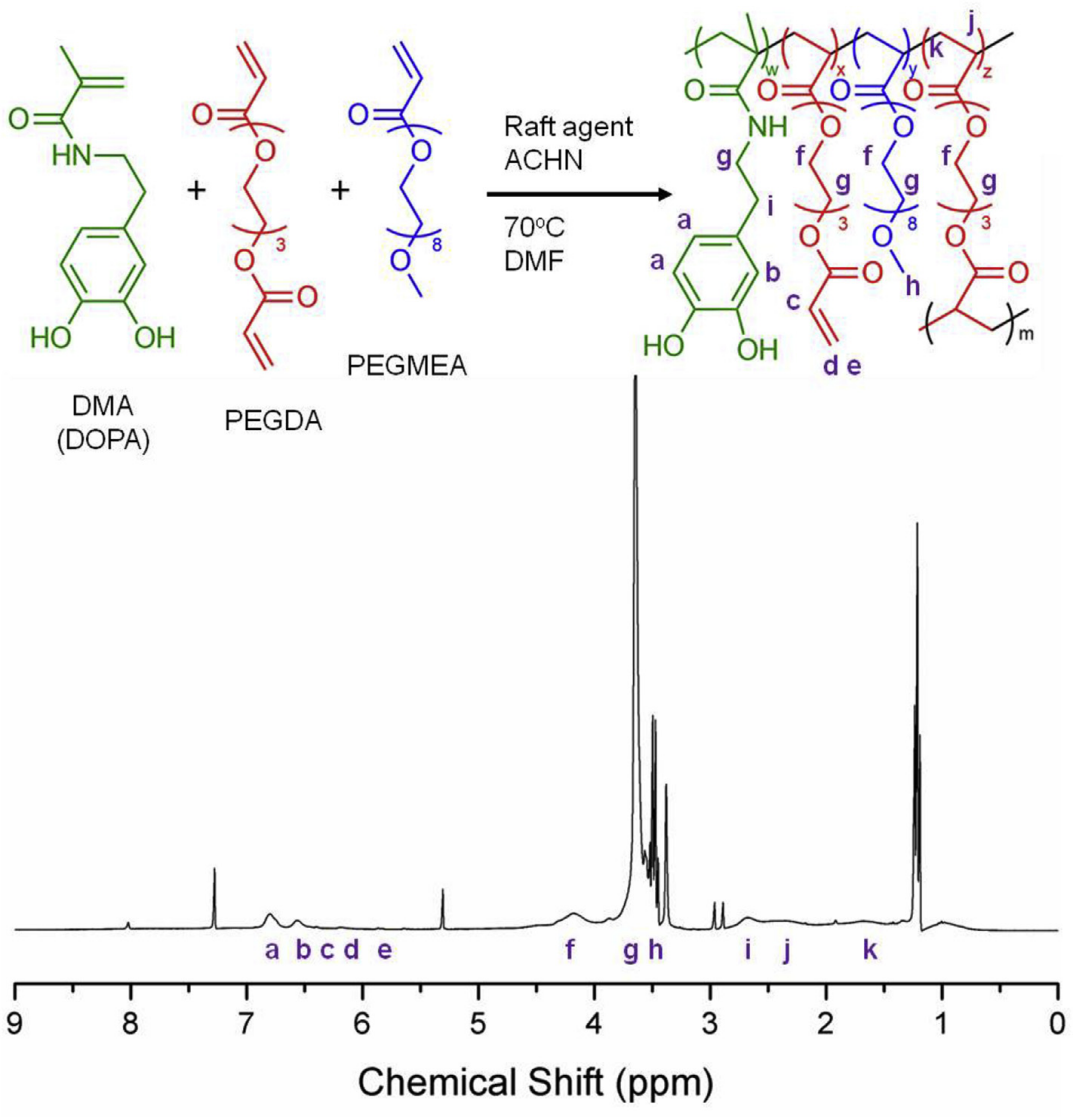
*Schematic depiction of RAFT polymerization with 1H NMR peak assignment*. RAFT co-polymerization of DMA, PEGDA and PEGMEA was carried out in DMF at 70 °C for 17 h to produce a DOPA containing polymer of Mw 18.8 kDa for anti-fibrillation applications.

Fig. 1 shows the reaction scheme for the formation of the DOPA/PEG containing polymer (DP) using three monomers. Dopamine methacrylamide (DMA) was used as the functional monomer due to its chatechol group, PEGMEA was used to introduce PEG into the structure (previously used for reducing toxicity), and PEGDA was the branching monomer due to the di-vinyl functionality. After 17 h reaction time, DP had a Mn of 11.75, Mw of 18.8 kDa, and a polydispersity index of 1.6. The feed ratio of DOPA to the PEG monomers was 40% and the final DOPA content calculated from the ^1^H NMR results (peak assignment shown in Fig. 1) was 42.8%. The amount of PEGDA involved in branching was 6.5% of the total monomer composition, with 4.6% present as free vinyl groups. The ease of synthesis and the ability to accurately adjust the amount of DOPA in the polymer structure makes RAFT polymerization an attractive strategy for the preparation of polymers for such anti-fibrillation applications. Furthermore, the presence of free-vinyl groups in the structure could allow functionalization such as the addition of antibodies or antibody fragments [31].

### 3.2 Effects of dopamine, PEG 12 and DP on α-synuclein aggregation

Earlier studies have shown that dopamine effectively promotes formation of oligomeric aggregates of α-synuclein [32], [33], [34], [35], [36]. These aggregates are resistant to further conversion to fibrils. This process involves oxidation of methionine residues of α-synuclein due to the ROS production by dopamine [37], [38]. In addition, oxidized dopamine can form covalent adducts with α-synuclein that further stabilize mostly disordered α-synuclein oligomers [39]. Covalent attachment of dopamine within the polymer structure was expected to interfere with the formation of covalent dopamine-α-synuclein adducts but should have at least partially preserved the effect of dopamine on α-synuclein aggregation due to the ROS formation.

Neutral polymers are known to promote macromolecular crowding, altering the kinetics of protein aggregation. For α-synuclein it has been shown [40], [41], [42] that compact, neutral polymers accelerate its aggregation at high concentrations (above 10 mg/mL) presumably by increasing effective protein concentration and stabilizing more compact protein conformations. Here we have included PEG 12 (polyethylene glycol, Mw 12 kDa) as a control to account for the effect of the polymer support itself on α-synuclein aggregation.

We examined the effects of moderate concentrations of our dopamine-modified polymer (1–20 mg/mL) as well as dopamine (0.5–10 mg/mL) and PEG 12 (1–20 mg/mL) on aggregation of α-synuclein. We used the aggregation conditions close to physiological that were previously shown to promote effective α-synuclein aggregation (0.25 mg/mL α-synuclein, 20 mM Hepes, pH 7.5, 0.1 M NaCl, 50 μg/mL heparin). Each experiment was run in triplicate, and independent experiments were performed at least 3 times for each data point.

**Fig. 2:**
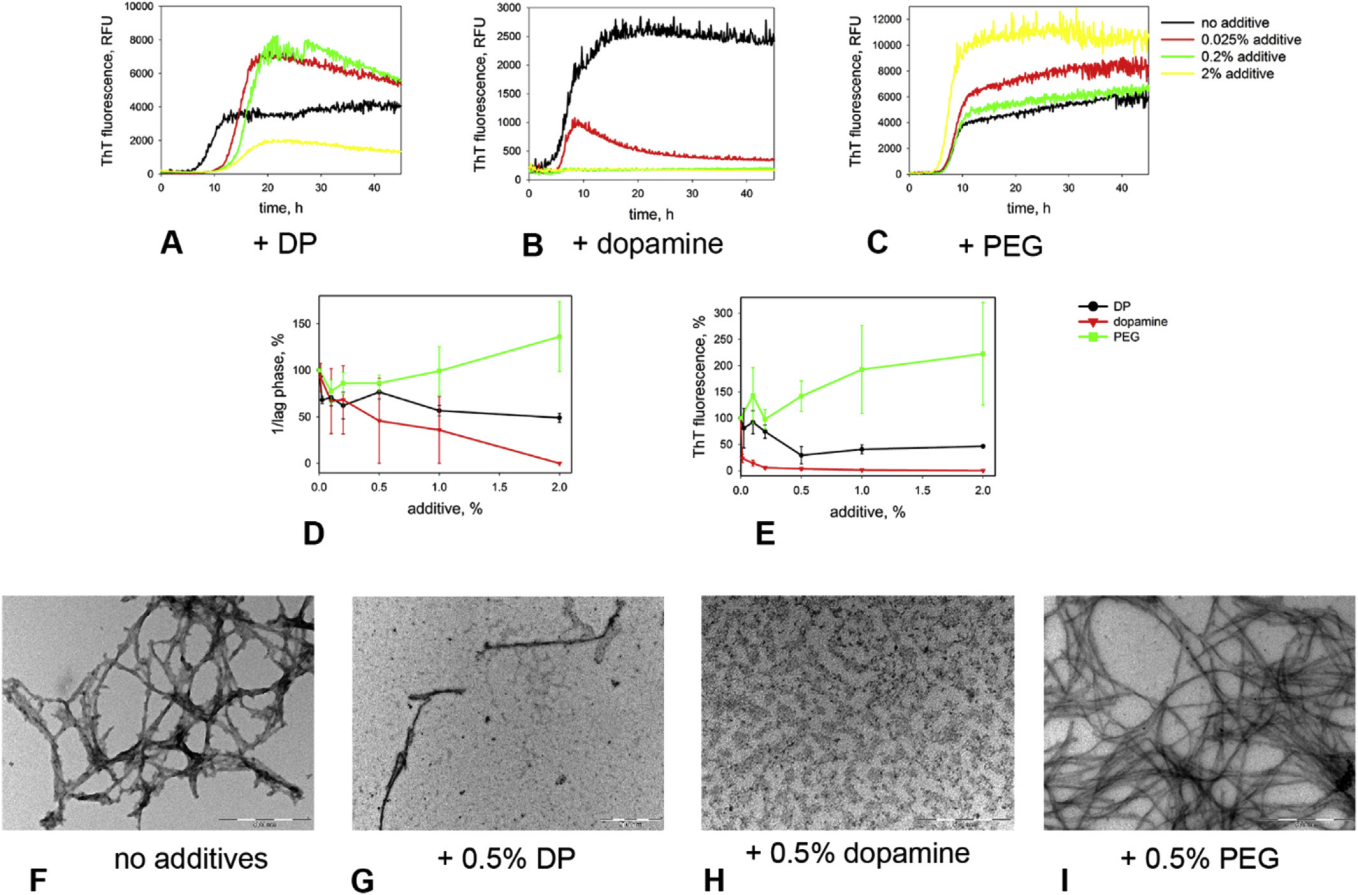
*Effect of additives on the kinetics of α-synuclein aggregation*. (A – C): Kinetic curves for fibril formation from α-synuclein (0.25 mg/mL, pH 7.5, 50 μg/mL heparin, 40 °C) in the presence of dopamine, dopamine-containing polymer (DP) and PEG 12. A – DP; B – dopamine; C PEG 12. Black – no additive, red–0.025% additive, green–0.2% additive, yellow–2% additive. (D–E): Initiation and elongation rates for fibril formation from α-synuclein in the presence of dopamine, DP and PEG 12. D – initiation rate (1/lag phase); E−fibril yield (ThT fluorescence). Black – dopamine, red – DP, green – PEG 12. Scale bars correspond to standard error between independent measurements. (F – I): EM images of aggregates obtained after 5 days of incubation. F – no additive; G – 0.5% DP; H – 0.5% dopamine; I – 0.5% PEG 12. Scale bars: 500 nm.(For interpretation of the references to colour in this figure legend, the reader is referred to the web version of this article.)

We found that DOPA-modified polymer inhibited formation of α-synuclein fibrils (Fig. 2 A–E). A longer lag phase was observed even at the lowest concentration tested (0.025%) while at higher concentrations (1–2%) the final levels of ThT fluorescence also decreased, indicating a smaller quantity of fibrils. However, the effect was modest with only ∼50% inhibition even at the highest concentrations tested. Electron microscopy (Fig. 2G) indicated that more oligomeric α-synuclein aggregates were formed in the presence of the polymer although fibrils were still present.

Dopamine itself was significantly more effective as an inhibitor of fibril formation. In the presence of dopamine the lag phase of α-synuclein aggregation increased and the ThT signal decreased significantly in the concentration-dependent manner. At dopamine concentrations of 10 mg/mL and higher, no increase in ThT fluorescence was observed indicating complete inhibition of fibril formation. Electron microscopy (Fig. 2H) confirmed these observations showing large quantities of oligomers and the absence of fibrils in the samples aggregates in the presence of dopamine.

PEG 12 accelerated α-synuclein aggregation decreasing the lag phase of aggregation somewhat and significantly increasing the ThT fluorescence indicating higher yield of fibrils (Fig. 2 A–E). And indeed electron microscopy (Fig. 2I) confirmed formation of large quantity of α-synuclein fibrils in the presence of PEG.

### 3.3 Disaggregation of α-synuclein fibrils by dopamine and the dopamine-containing polymer

In addition to interfering with formation of protein fibrils, some compounds are known to disaggregate the preformed fibrils. A peculiar shape of α-synuclein aggregation curves in the presence of dopamine (Fig. 2B) consisting of the sigmoidal rise of ThT fluorescence followed by exponential decrease suggested that dopamine might be disaggregating α-synuclein fibrils. We decided to examine whether this is indeed the case.

**Fig. 3:**
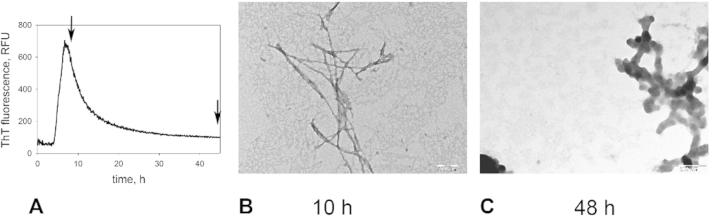
*Morphology of α-synuclein aggregates formed in the presence of dopamine at different time points*. A – kinetic curve of α-synuclein aggregation in the presence of 0.1% dopamine; B – aggregates at 10 h; C – ggregates at 48 h. Time points are marked with arrows. Scale bars: 200 nm.

**Fig. 4:**
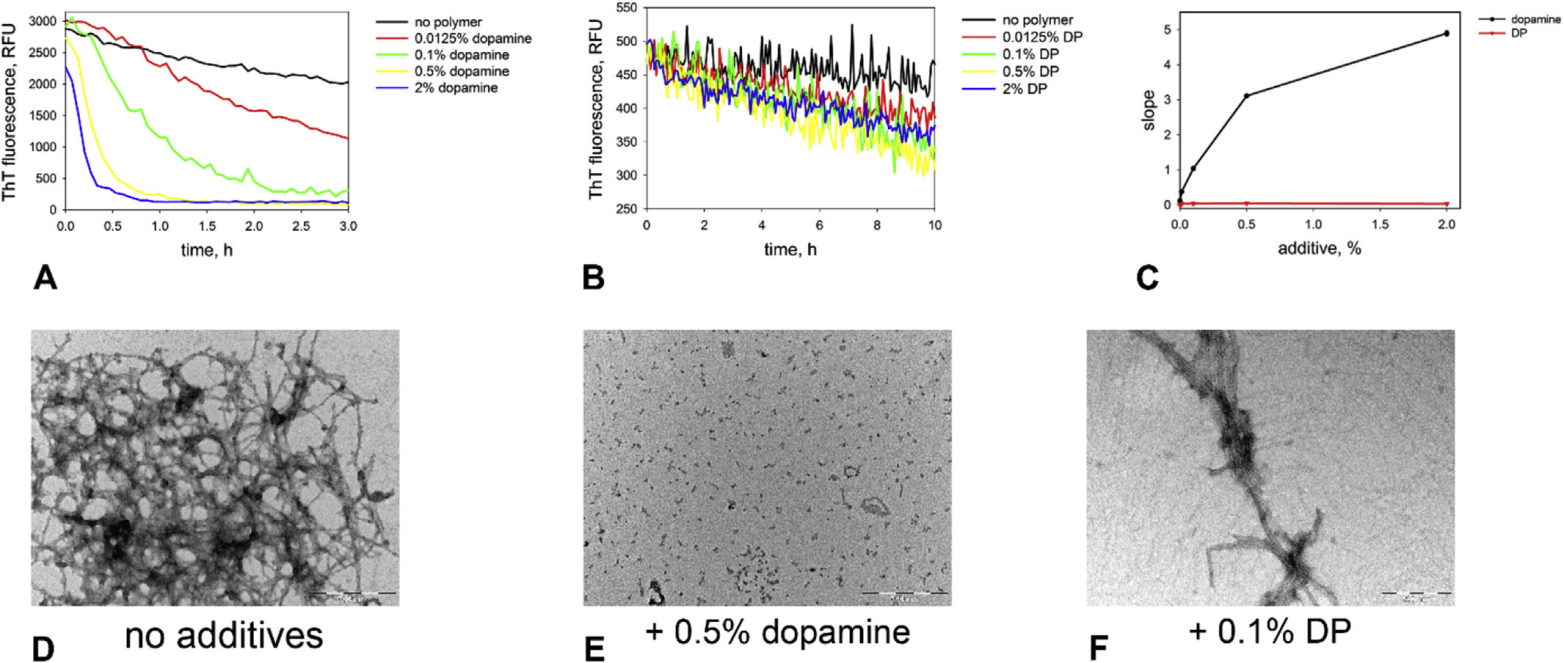
*Disaggregation of α-synuclein fibrils by dopamine and DP*. A, B – kinetic curves for disaggregation of preformed α-synuclein fibrils (0.05 mg/mL, pH 7.5, 50 μg/mL heparin, 40 °C) in the presence of dopamine or DP. A – dopamine; B – DP. Black – no additive, red–0.025% additive, green–0.5% additive, yellow–2% additive. C – exponential decay rate of ThT fluorescence of α-synuclein fibrils incubated in the presence of DOPA and DP. D – E: morphology of fibrils after incubation with the additive for 15 h. D – no additives, E 0.5% dopamine, F – 0.1% DP.(For interpretation of the references to colour in this figure legend, the reader is referred to the web version of this article.)

We analyzed the morphology of α-synuclein aggregates by electron microscopy at different time points during aggregation in the presence of dopamine indicated by arrows in Fig. 3A. We found that at the endpoint of aggregation, only oligomeric aggregates were present (Fig. 3C). However, at the intermediate point during the aggregation (after the sigmoidal rise in ThT fluorescence but before its subsequent exponential drop) primarily amyloid fibrils were observed (Fig. 3B). This result strongly suggests that dopamine (or a product of dopamine oxidation) is highly effective in disaggregating preformed α-synuclein amyloid fibrils. We have also tested this directly by incubating α-synuclein fibrils (0.05 mg/mL) in the presence of dopamine (Fig. 4A). We found that dopamine rapidly disaggregates the fibrils in concentration-dependent manner. The process of disaggregation is complete in minutes at higher dopamine concentrations. We have also tested the ability of DP to disaggregate the α-synuclein fibrils in a similar fashion (Fig. 4B). We found a weak effect of the polymer on ThT fluorescence of the fibrils indicating that DP does mediate fibril disaggregation, but to a significantly lesser extent than dopamine. Electron microscopy (Fig. 4D–F) confirmed that α-synuclein fibrils completely disaggregated into oligomeric aggregates in the presence of dopamine but remained largely intact in the presence of DP.

Since PD pathology is associated with dopaminergic neurons, interaction between α-synuclein and dopamine has been extensively investigated [37]. Dopamine is known to bind to α-synuclein non-covalently inhibiting its fibrillation and stabilizing the oligomers [43]. However, dopamine is highly susceptible to oxidation and its oxidation products form adducts with α-synuclein [39], [44]. These adducts drive aggregation of α-synuclein into primarily unstructured, sodium dodecyl sulfate (SDS)-resistant oligomers [39], [44], [45]. Therefore, dopamine interferes with protein aggregation via a variety of pathways. Most of these pathways require either direct protein–dopamine interaction or proximity between dopamine and a protein molecule. Incorporation of dopamine within a PEG polymer was expected to result in a large molecule capable of inhibiting fibrillation. This was achieved but to a lesser extent than free dopamine due to its immobilization interfering with dopamine–protein interactions.

Overall, we synthesized a dopamine-containing hyperbranched polymer via RAFT co-polymerization with PEG monomers. We found that, similar to dopamine itself, the polymer interfered with the α-synuclein fibril formation promoting oligomer formation instead. However, effects of dopamine were significantly moderated by its incorporation into a polymer, and it was no longer capable of effectively disaggregating fibrils into oligomers. This is likely due to a specific mechanism of action of dopamine known to involve covalent interaction with the target proteins. The ease at which the polymer structure and composition can be varied allows the potential to mechanistically study specific polymer/protein interactions in an attempt to find future therapeutic strategies for diseases associated with protein misfolding.
